# NCAPD3 is involved in papillary thyroid carcinoma proliferation, metastasis, and aerobic glycolytic pathway

**DOI:** 10.1007/s12672-025-02767-x

**Published:** 2025-05-30

**Authors:** Liubing Zhang, Aiping Peng, Yue Qin

**Affiliations:** 1 Department of Medical Laboratory, Longhua District People’s Hospital, Shenzhen, 518100 China; 2 Department of Nephrology, Longhua District People’s Hospital, Shenzhen, 518100 China; 3 Department of Pathology, Longhua District People’s Hospital, Shenzhen, 518100 China

**Keywords:** NCAPD3, Papillary thyroid carcinoma, Proliferation, Metastasis, Aerobic glycolysis

## Abstract

**Objective:**

To examine the expression of non-SMC condensin II complex subunit D3 (NCAPD3) in papillary thyroid carcinoma (PTC) tissues, assess its impact on the growth and metastatic potential of PTC cells, and investigate its role in regulating glycolysis to uncover the underlying mechanisms involved.

**Methods:**

NCAPD3 levels in PTC tissues were detected using immunohistochemistry. siRNA transfection was used to silence NCAPD3 in K1 and TPC-1 cells. Cell proliferation rates were detected using the Cell Counting Kit-8 assay, migration rates were evaluated using wound healing and Transwell cell migration assays, and invasion rates were assessed using the Transwell-Matrigel cell invasion assay. Moreover, the aerobic glycolysis-related factors lactate, lactate dehydrogenase A (LDHA), and pyruvate kinase M2 (PKM2) were detected using kits.

**Results:**

NCAPD3 was highly expressed in PTC tissues. Its expression showed no significant association with patient age, gender, or lymphocytic thyroiditis but was significantly correlated with larger tumor size and lymphovascular invasion. NCAPD3 expression significantly decreased in K1 and TPC-1 cells after transfection with siRNA. Low NCAPD3 expression reduced the proliferation rate of K1 and TPC-1 cells and inhibited cell migration and invasion. Moreover, NCAPD3 silencing decreased LDHA, PKM2, and lactate levels.

**Conclusions:**

NCAPD3 was highly expressed in PTC tissues, and correlated with aggressive features (tumor size and lymphovascular invasion). NCAPD3 silencing inhibited proliferation, migration, invasion, and aerobic glycolysis of PTC cells. Therefore, NCAPD3 may serve as a potential therapeutic target for PTC.

**Supplementary Information:**

The online version contains supplementary material available at 10.1007/s12672-025-02767-x.

## Introduction

Thyroid cancer is the ninth most common malignant tumor worldwide and its incidence has increased over the past few decades [1]. Papillary thyroid carcinoma (PTC) is the most common type of thyroid cancer, accounting for approximately 90% of all cases. The prognosis of most patients with PTC is favorable, with a 5-year survival rate of > 98% [2]. However, the onset of PTC is occult and approximately 35% of patients have external thyroid invasion or distant metastasis at the time of diagnosis, resulting in a relatively poor prognosis [3]. Therefore, clarifying the molecular mechanism behind the potential malignant behavior of PTC can provide a new approach for targeted intervention therapy.

Condensin is a chromatin-binding protein that regulates chromatin coagulation and stabilization during mitosis. Condensins are involved in chromosome assembly and are essential for reproduction. Most eukaryotes contain two types of condensins, condensin I and condensing II. The non-SMC condensin II complex subunit D3 (NCAPD3) is one of the condensin II subunits [4]. Abnormal NCAPD3 function may lead to the interruption of chromosome condensation, which affects the process of cell mitosis. Increasing evidences show that NCAPD3 also involves in the occurrence and development of tumors. NCAPD3 is high-expressed in prostate cancer, gastric cancer, non-small cell lung cancer, and colorectal cancer tissues [5–9]. Its expression is associated with a poor prognosis of patients with gastric cancer, colorectal cancer, and [7–9]. Functional experiments in vitro demonstrated that NCAPD3 exerts a pro-tumor effect in prostate cancer, gastric cancer, non-small cell lung cancer, and colorectal cancer [5–9]. However, the role of NCAPD3 in thyroid cancer remains unknown.

In this study, we collected PTC and adjacent normal tissues to detect the expression of NCAPD3. Using K1 and TPC-1 cells, we investigated the effects of NCAPD3 silencing on the proliferation, migration, and invasion of PTC in vitro. We also investigated the effect of NCAPD3 on aerobic glycolysis to explore its potential mechanism in PTC cells. This evidence provides a basis for the clinical treatment of PTC.

## Material and methods

### Sample collection

Twenty patients with PTC at Longhua District People’s Hospital were selected for the study from February 2023 to November 2023, including 8 males and 12 females. Age ranged from 21 to 69 years (mean age 50.91 ± 14.74 years). All patients had pathologically confirmed PTC, according to the Chinese Expert Consensus on the Diagnosis and Treatment of Thyroid Microcarcinoma (2016 edition). The TNM stages ranged from I to IV. These patients had never received preoperative radiotherapy or chemotherapy. Patients with other malignancies, thyroid diseases, or recent use of drugs affecting thyroid function were excluded. All the patients who participated in the study provided written informed consent. This study was approved by the Medical Ethics Committee of Longhua District People’s Hospital (No.2024–098) and conducted in accordance with the Declaration of Helsinki.

### Immunohistochemical (IHC) and analysis

The tissue specimens were fixed in 4% paraformaldehyde, treated with gradient dehydration with ethanol, then embedded in paraffin, and cut into sections with a thickness of 4 μm. The sections were transferred onto slides, dried, and melted. The antigens were then repaired using a citrate buffer. The slides were soaked in Tris-buffered saline (TBS) for 3 min. The cells were treated with 3% hydrogen peroxide at room temperature for 30 min. After washing with TBS for 3 min, the slides were incubated with 10% goat serum for 30 min at room temperature. The slides were incubated with NCAPD3 antibody (1:50, Signalway Antibody LLC, Greenbelt, MD, USA) overnight at 4℃. The next day, the slides were washed with TBST. They were then incubated with a goat anti-rabbit IgG secondary antibody. Afterwards, the slides were washed with TBST and treated with 50 μL freashly prepared DAB solution, and water was added to stop the reaction. Nuclei were stained with hematoxylin for 1 min and then differentiated using hydrochloric acid alcohol for 1 s. After washing, bluing solution was applied for a few seconds. The slides were observed under a light microscope, and the results were recorded. The IHC results were independently reviewed by two observers. Staining intensity (negative = 0, light yellow = 1, brown = 2, brown = 3) and staining degree (positive cells as a percentage of the total counted cells, < 25% = 1, 26%−50% = 2, 51%−75% = 3, > 75% = 4) were assessed. The product of the staining intensity and staining degree was used as the total score of NCAPD3 staining. Twenty PTC patients were stratified into two groups based on the total score of NCAPD3 staining: the NCAPD3 low-expression group (total score ≤ 4) and the NCAPD3 high-expression group (total score > 4). Fisher’s exact test was used to analyze the association between NCAPD3 expression levels and clinicopathological characteristics.

### Cell culture and transfection

The K1 and TPC-1 cells were purchased from Ningbo Mingzhou Biotechnology Co., LTD (Ningbo, China). K1 cells were cultured in DMEM-F12 K supplemented with 10% fetal bovine serum (FBS). TPC-1 cells were cultured in RPMI 1640 medium supplemented with 10% FBS. All cells were cultured at 37℃ with 5% CO2 in humidified atmosphere. Negative control small interfering RNA (siRNA) (NC group) and two siRNAs targeting NCAPD3 (siRNA1 and siRNA2 groups) were transfected into cells using Lipofectamine 2000 (Cat. No. 11668019, Invitrogen, Carlsbad, CA, USA) according to the manufacturer’s instructions.

### qRT-PCR

After transfection for 48 h, the total RNA of each group of cells was extracted using TRIzol (15596026 CN, Invitrogen) and quantified using a biophotometer plus (Agilent Technologies, Santa Clara, CA, USA). Total RNA was reverse transcribed into cDNA using a Reverse Transcriptase Kit (Vazyme, Nanjing, China). qRT-PCR was performed using SYBR Green qPCR Master Mix (Q111-02; Vazyme). The relative expression of NCAPD3 was calculated using the 2^−ΔΔCT^ method. The 18 s rRNA was used as an internal control. The primers were synthesized by Takara Bio. The primers for NCAPD3 and 18 s were as follows: NCAPD3-F, AAGGGCCACGTGAGTAAAG; NCAPD3-R, CTGCCTCTTCCTGAGATGAC; 18 s-F, CCTGGATACCGCAGCTAGGA; 18 s-R, GCGGCGCAATACGAATGCCCC.

### Western blotting (WB)

After culturing for 48 h, the cells were lysed using a cell lysis solution (Beyotime, Shanghai, China) containing a protease inhibitor and PMSF. After cell lysis, the cells were centrifuged at 14,000 rpm at 4 ℃ for 5 min to remove cell debris, and the supernatant protein solution was mixed with PMSF and stored at −20 ℃. Total protein was quantified using a bicinchoninic acid (BCA) protein assay kit (Thermo Scientific Pierce, Rockford, IL, USA). A total of 30 μg of protein samples were separated using SDS-PAGE and were transferred onto a polyvinylidene difluoride (PVDF) membrane (Pall, New York, NY, USA). Non-fat milk was used to enclose the PVDF membrane at room temperature for 2 h. After washing thrice with TBST, the primary antibody (Signalway Antibody LLC) was added and incubated at 4℃ overnight. The primary antibody included NCAPD3 (1:1000) and anti-GAPDH (1:2000). After washing three times with TBST, the Rabbit anti-mouse IgG (H + L)-HRP secondary antibody (Cell Signaling Technologies, Danvers, MA, USA) was added to the membrane and incubated at room temperature for 2 h. Finally, the membrane was washed three times with TBST, and the chemical fluorescent substrate was added for 1–5 min. Excess solution was removed from the PVDF membrane and developed using an automatic gel imager (Bio-Rad, Hercules, CA, USA). Using GAPDH as an internal reference, the gray values of each strip were quantitatively analyzed using ImageJ software (Media Cybernetics, Rockville, MD, USA), and the relative expression level of the NCAPD3 protein was calculated.

### Cell counting kit-8 (CCK-8) assay

After transfection, the cells were adjusted to 10^5^/mL and were inoculated with 10^4^ cells per well in 96-well culture plates. K1 and TCP-1 cells were incubation for four days. Cell proliferation was assessed using a CCK-8 solution (Dojindo, Japan). In brief, 100 μL of fresh DMEM without FBS and 10 μL of CCK-8 solution were added to each well, followed by incubation for 1 h at 37 ℃. The optical density (OD) was measured using a microplate reader (Multiscan MK3; Thermo Fisher Scientific, Waltham, MA, USA) at 490 nm.

### Wound healing assay

After transfection for 24 h, the cell density was adjusted to 10^5^/mL and inoculated into 6-well plates. When the cell density reached 95%, cells were scratched with a 10 μL pipette tip of a sterile gun. After washing twice with PBS, the medium was replaced with serum-free medium. Cells in the same position were photographed at 0 h, 24 h, and 48 h. The distance between the cells was measured, and the average scratch healing rate was calculated as follows: average scratch healing rate = (0 h scratch width −24 h/48 h scratch width)/0 h scratch width × 100%.

### Transwell cell migration assay

After transfection for 24 h, the transwell assays were used to measure migration and invasion. Cell density was adjusted to 10^5^/mL. Cell migration assay: A total of 1 × 105 cells from each group were resuspended in serum-free medium and seeded into the upper chamber. The complete medium was added to the lower chamber. After 24 h of incubation, the cells in the upper chamber were removed using cotton swabs. Cells on the membrane were fixed with 4% paraformaldehyde for 15 min and stained with 0.1% crystal violet. Five fields of view were randomly selected and imaged using a DMI4000B microscope (Leica, Wetzlar, Germany). Number of migrated cells in each field was counted.

### Transwell-Matrigel cell invasion assay

The invasion rate was determined using the Transwell–Matrigel assay. The Transwell Matrigel assay was similar to the Transwell assay, except that the Transwell Matrigel assay required precoating with Matrigel in the upper chamber. The Matrigel was dissolved at 4 ℃ and then dilluted with serum-free medium in a ratio of 1:3. A total of 40 μL of the diluted Matrigel was added into the upper chamber, then incubated at 37℃ for 2 h to solidify. The excess liquid was removed from the chamber, the cells were seeded in the upper chamber, and 600 μL serum-free medium was added to the lower chamber. The system was incubated for 24 h, then the cells that invaded the Matrigel were assessed, and images were captured. Number of invasive cells in each field was counted.

### Detection of LDHA, PKM2, and lactate in medium supernatants

After transfection for 48 h, the K1 and TCP-1 cells in each group and their culture medium supernatants were collected. The levels of LDHA and PKM2 in the cell lysates were detected using LDHA ELISA kits (KL-PKM2-Hu, Kanglang Biology, Shanghai, China) and PKM2 ELISA kits (E-EL-H0556, Elabscience, China), respectively. Lactate levels in the supernatants were measured using a Lactate Assay Kit (KTB1100; Abbkine, Wuhan, China), according to the manufacturer’s instructions.

### Statistical analysis

The results are presented as mean ± standard deviation. Paired t-tests and one-way analysis of variance (ANOVA) were used to analyze the data using GraphPad Prism 9.0 software (GraphPad Software, San Diego, CA, USA). Statistical significance was set at P < 0.05.

## Results

### NCAPD3 expression in PTC tissues

**Fig. 1:**
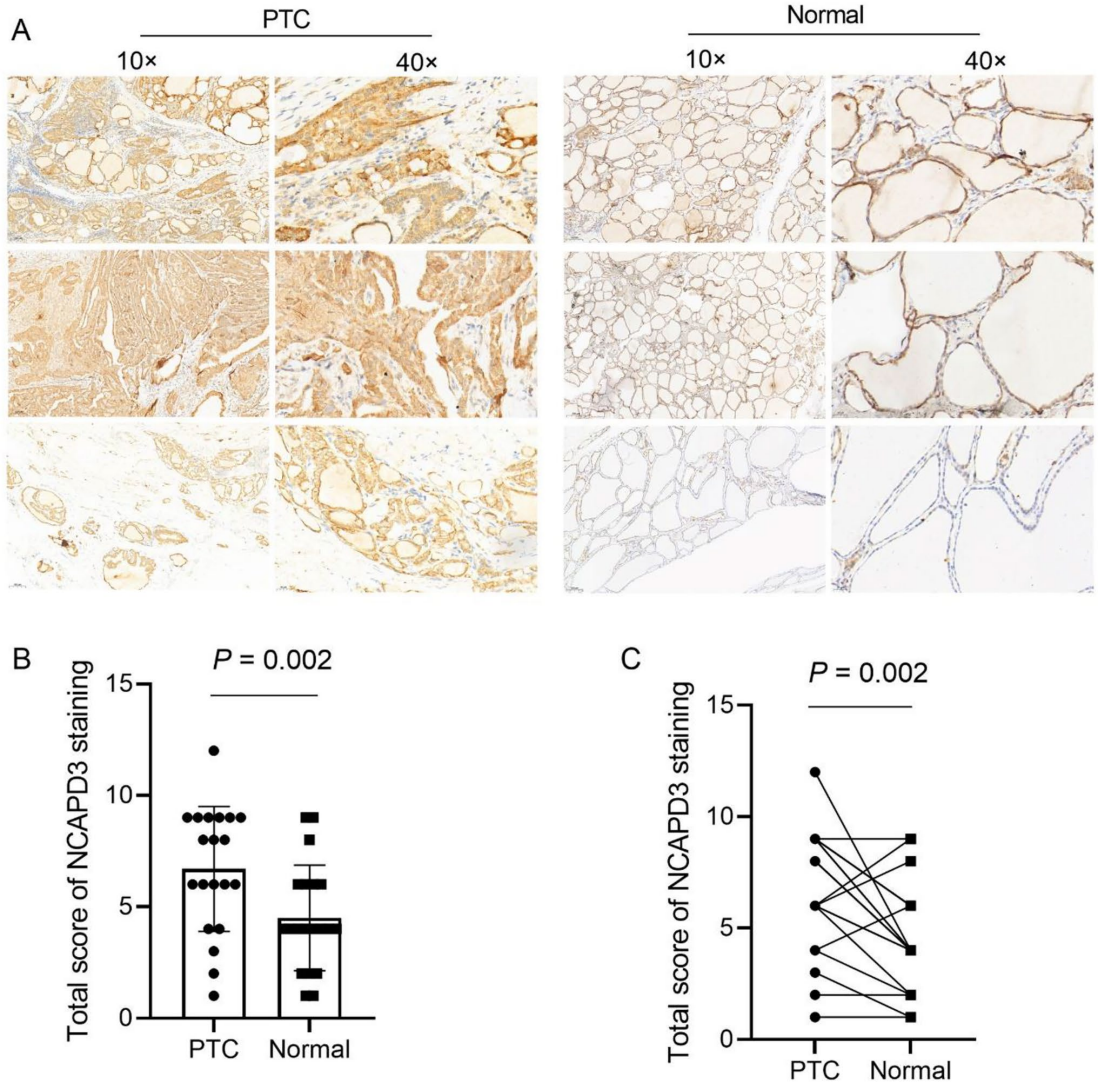
NCAPD3 expression in PTC and adjacent normal tissues. **A** Images showing the expression of NCAPD3. **B** Analysis of total NCAPD3 expression scores in PTC and adjacent normal tissues. **C** Paired analysis of total NCAPD3 expression scores in PTC and corresponding adjacent normal tissues

IHC was used to analyze NCAPD3 levels in 20 PTC samples and adjacent normal tissues and representative images of three cases are presented in Fig. 1A. NCAPD3 expression was positive in all samples. The total score of NCAPD3 in PTC tissues was significantly higher than that of adjacent normal tissues [(6.70 ± 2.81) vs. (4.50 ± 2.37), *t* = 3.609, *P* = 0.002] (Fig. 1B). Paired analysis showed that NCAPD3 expression higher in 14 PTC tissues, lower in 3, and remained unchanged in 3 relative to adjacent normal tissues (Fig. 1C). Furthermore, we analyzed the relationship between NCAPD3 expression level and the clinical characteristics of patients with PTC. As shown in Table 1, NCAPD3 expression in PTC tissues showed no significant association with patient age, gender, or lymphocytic thyroiditis, but was significantly correlated with tumor size and lymphovascular invasion.

**Table 1:** Correlation between NCAPD3 protein levels and clinicopathological characteristics of patients with PTC

Characteristic	n	NCAPD3 protein levels		P value
		Low	High	
Age (years)				
< 60	13	3	10	> 0.999
≥ 60	7	2	5	
Gender				> 0.999
Male	8	2	6	
Female	12	3	9	
Tumor size (cm)				0.031
< 1	7	4	3	
≥ 1	13	1	12	
TNM stage				0.530
I + II	16	5	11	
III + IV	4	0	4	
Lymphovascular invasion				0.038
No	11	5	6	
Yes	9	0	9	
Lymphocytic thyroiditis				> 0.999
Absent	14	4	10	
Present	6	1	5	

### Expression of NCAPD3 after siRNA transfection

**Fig. 2:**
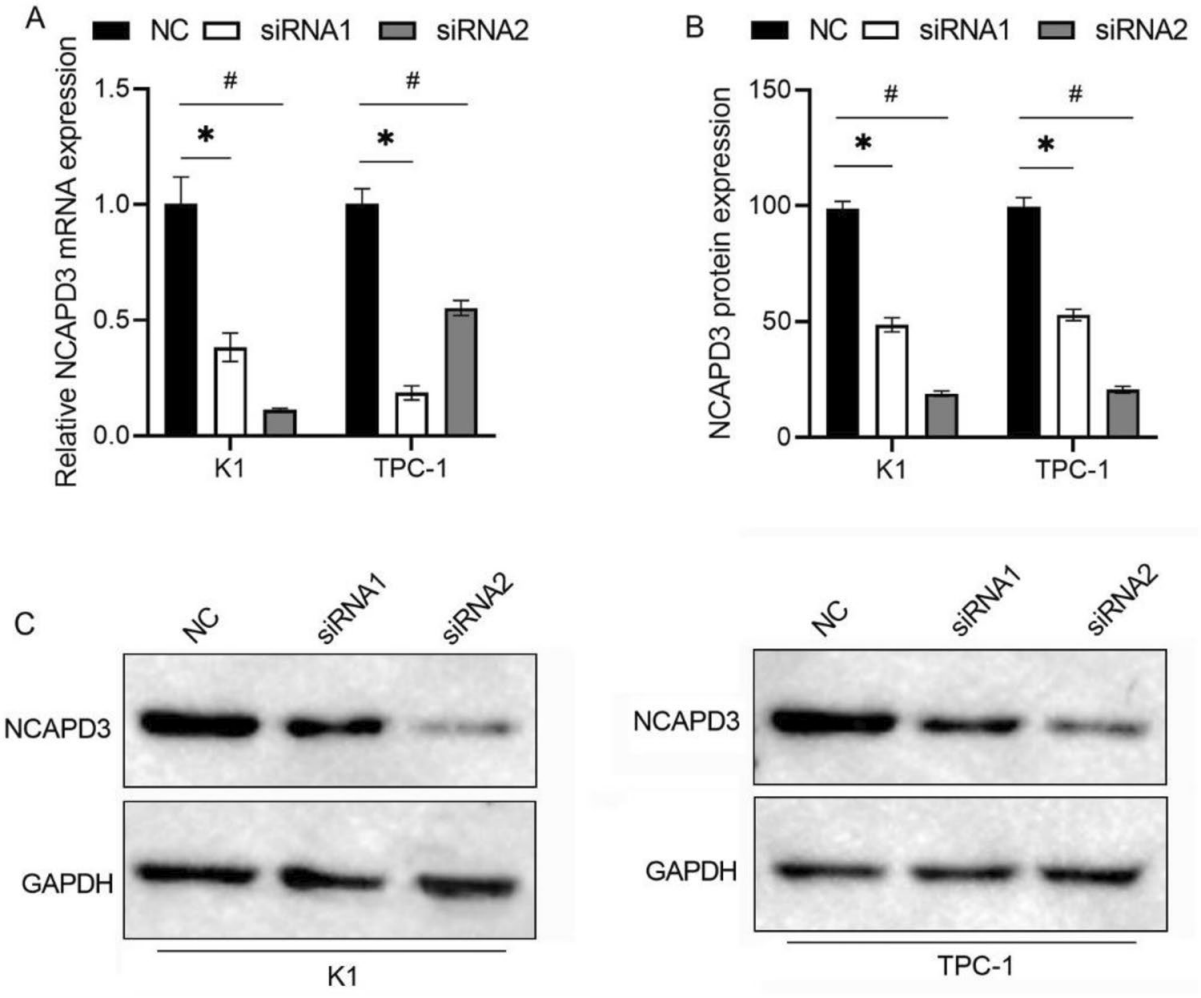
NCAPD3 expression level in K1 and TPC-1 cells after transfection with siRNA. Transfection with siRNA1 and siRNA2 reduced NCAPD3 mRNA (**A**) and protein (**B**, **C**) expression levels. Panel B is the bar graph on NCAPD3 expression level normalized to reference protein GAPDH based on the gray values of each band. * P < 0.05, siRNA1 vs. NC; # P < 0.05, siRNA2 vs. NC

NCAPD3 expression was knocked down by siRNA transfection. qRT-PCR and western blotting were used to determine the transfection efficiency. As shown by qRT-PCR (Fig. 2A) and WB (Fig. 2B and C) results, NCAPD3 mRNA and protein expression in both the siRNA1 and siRNA2 groups were significantly reduced compared to those in the NC group (*P* < 0.05). These results indicated that the transfection with siRNA1 and siRNA2 successfully knocked down NCAPD3 expression.

### NCAPD3 silencing on cell proliferation in vitro

**Fig. 3:**
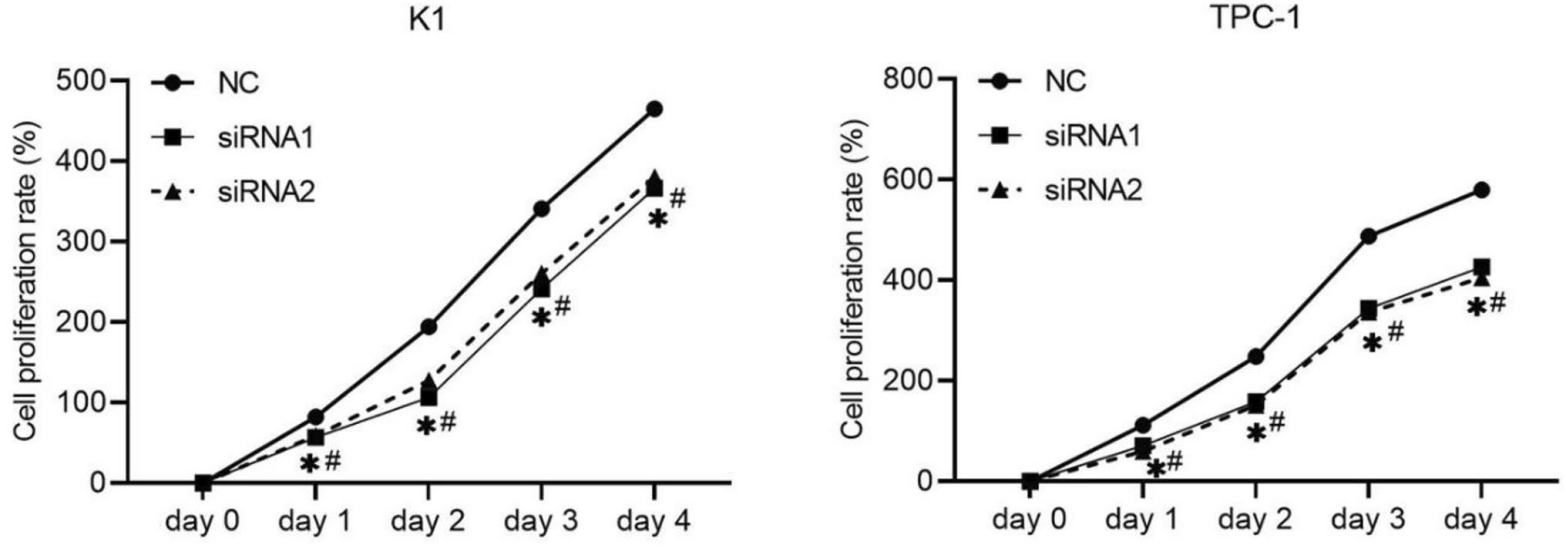
Effect of NCAPD3 silencing on cell proliferation. K1 and TPC-1 cells were transfected with NC siRNA and two siRNA of NCAPD3 (siRNA1 or siRNA2). After transfection, CCK-8 assay was performed and cell proliferation rate was calculated according to the results of CCK-8 assay. * P < 0.05, siRNA1 vs. NC; # P < 0.05, siRNA2 vs. NC

The effect of NCAPD3 silencing on the proliferation of K1 and TPC-1 cells was tested using a CCK-8 assay. Cell proliferation rate in both the siRNA1 and siRNA2 groups was lower than that in the NC group on day 2, day 3 and day 4 (Fig. 3). These results suggest that NCAPD3 silencing can inhibit PTC cell proliferation.

### NCAPD3 silencing inhibited PTC cell migration and invasion

**Fig. 4:**
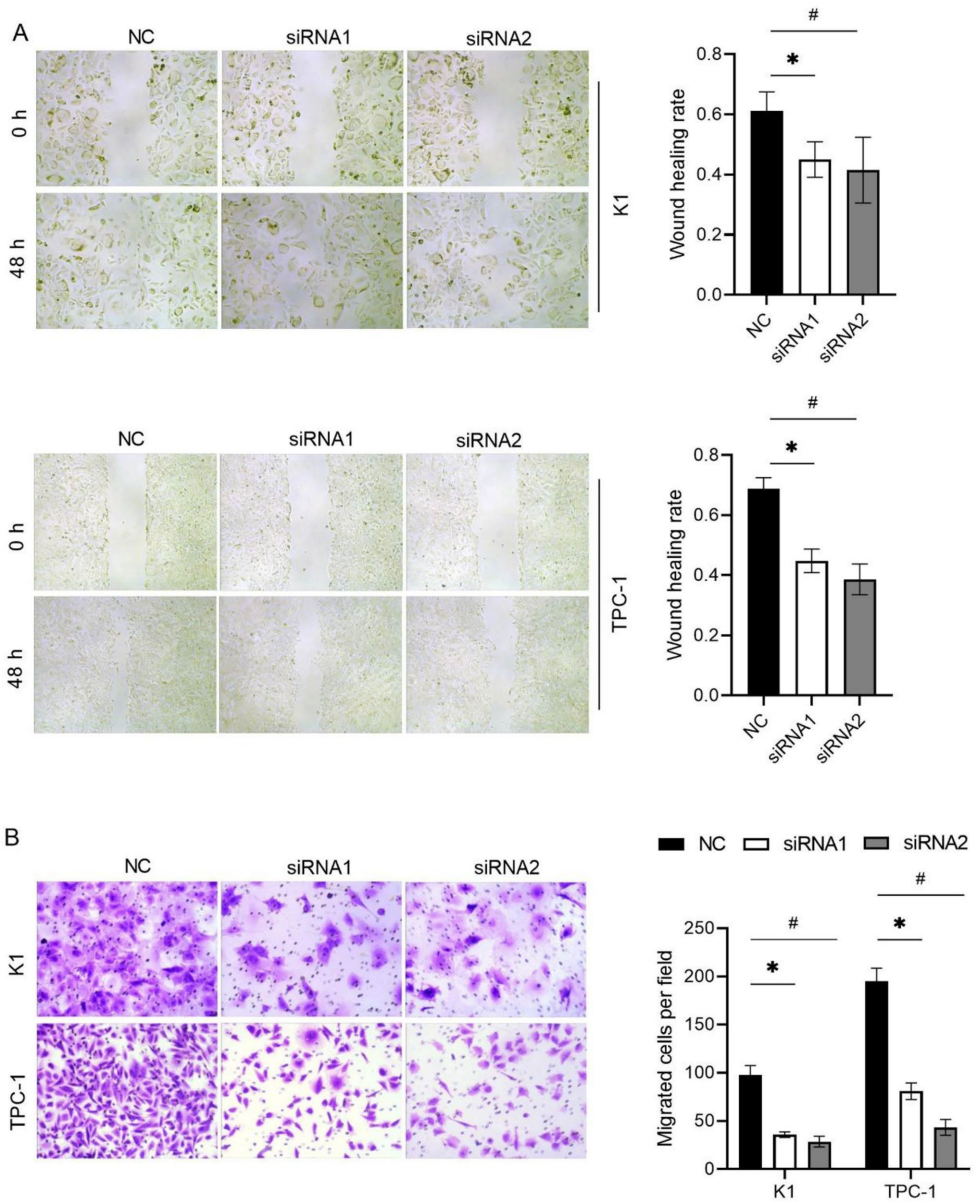
Effect of NCAPD3 silencing on cell migration. K1 and TPC-1 cells were transfected with NC siRNA and two siRNA of NCAPD3 (siRNA1 or siRNA2). After transfection, wound healing (**A**) and Transwell cell migration (**B**) assays were performed to assess cell migration capability. * P < 0.05, siRNA1 vs. NC; # P < 0.05, siRNA2 vs. NC

**Fig. 5:**
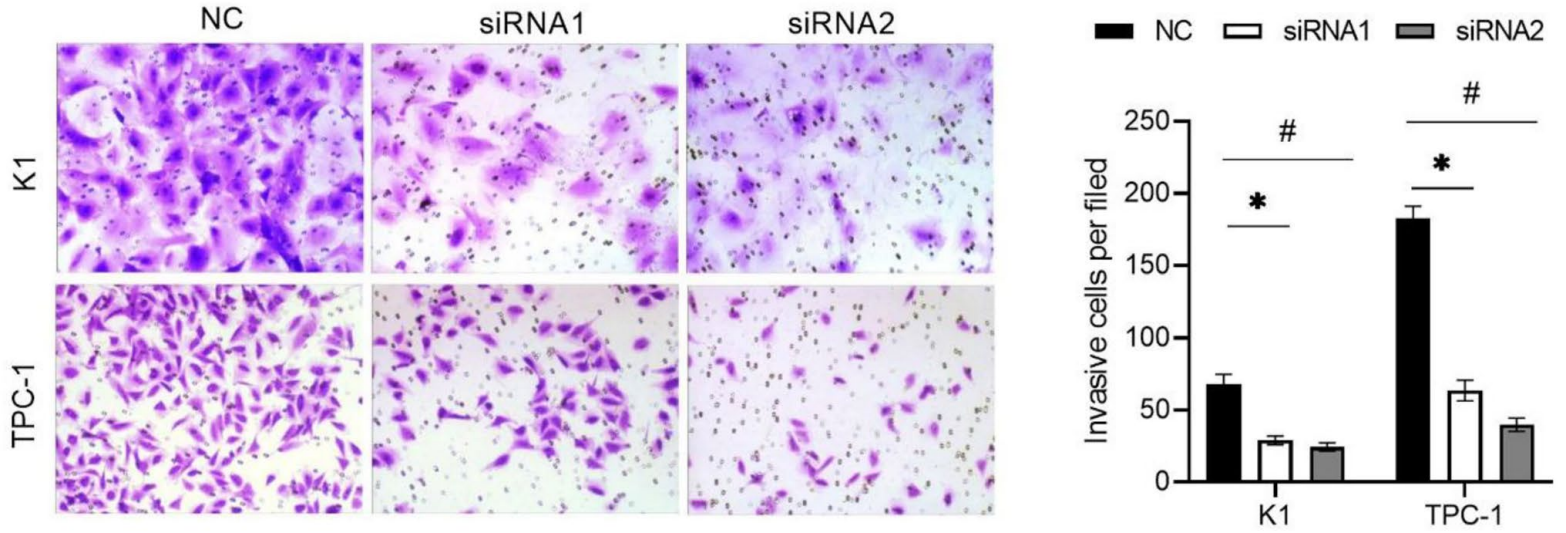
Effect of NCAPD3 silencing on the cell invasion. K1 and TPC-1 cells were transfected with NC siRNA and two siRNA of NCAPD3 (siRNA1 or siRNA2). After transfection, Transwell-Matrigel cell invasion assay was carried out and the number of invasive cells was calculated. * P < 0.05, siRNA1 vs. NC; # P < 0.05, siRNA2 vs. NC

The effect of NCAPD3 silencing on the migration of K1 and TPC-1 cells was tested using wound healing and Transwell cell migration assays. As shown by the wound healing assay (Fig. 4A), the wound healing rate in both the siRNA1 and siRNA2 groups was lower than that in the NC group. Moreover, the number of migrated cells in both the siRNA-1 and siRNA-2 groups was significantly reduced compared to that in the NC groups (Fig. 4B). The effect of NCAPD3 silencing on the invasion of K1 and TPC-1 cells was tested using the Transwell-Matrigel cell invasion assay. The results showed that the number of invasive cells in both siRNA1 and siRNA2 groups was significantly reduced compared to that in the NC groups (*P* < 0.05) (Fig. 5). These results suggest that NCAPD3 silencing inhibited PTC cell migration and invasion.

### NCAPD3 silencing inhibited the levels of LDHA, PKM2, and lactate in PTC cells

**Fig. 6:**
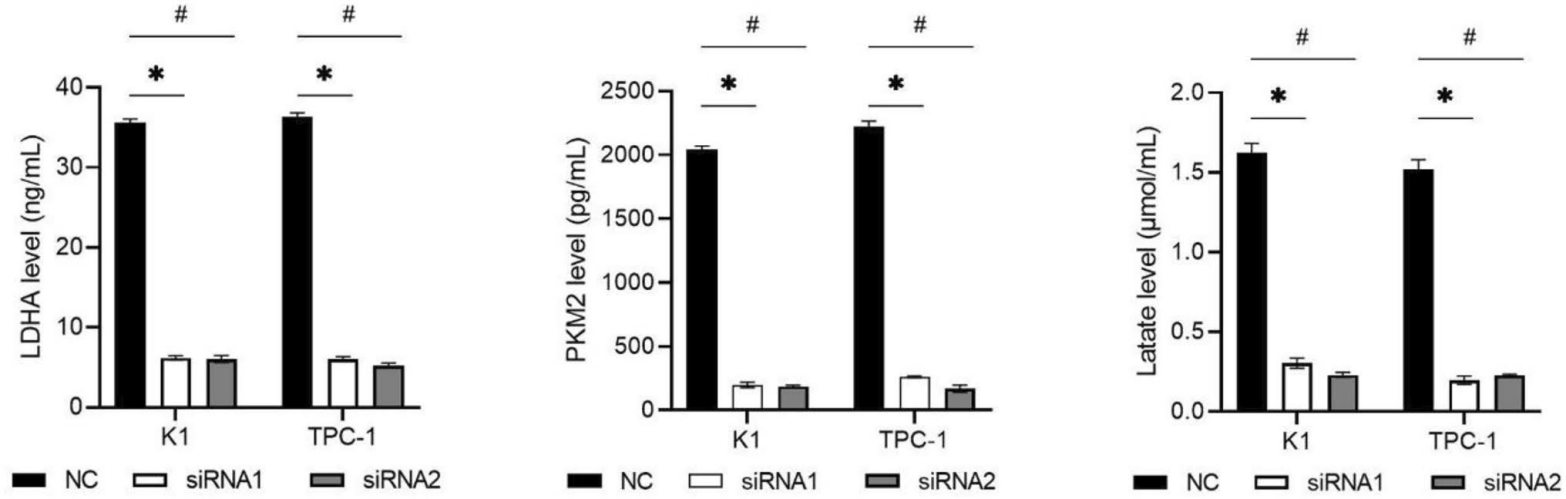
Effect of NCAPD3 silencing on the levels of LDHA, PKM2, and lactate in K1 and TPC-1 cells. K1 and TPC-1 cells were transfected with NC siRNA and two siRNA of NCAPD3 (siRNA1 or siRNA2). After transfection, levels of LDHA and PKM2 in cell lysates and lactate level in culture medium supernatants were measured. * P < 0.05, siRNA1 vs. NC; # P < 0.05, siRNA2 vs. NC

The aerobic glycolysis pathway plays an important role in tumor development and is closely related to tumor growth and recurrence [10]. LDHA and PKM2 are key enzymes in aerobic glycolysis and lactate is the product of glycolysis [10]. To investigate the effect of NCAPD3 on the aerobic glycolysis pathway in PTC cells, LDHA, PKM2, and lactate levels were measured. The levels of LDHA, PKM2, and LA in both siRNA-1 and siRNA-2 groups were lower than those in the NC groups (Fig. 6). These results indicated that NCAPD3 silencing can inhibit the aerobic glycolysis pathway in PTC cells.

## Discussion

Our present study showed that NCAPD3 protein expression was significantly higher in PTC tissues than in adjacent normal tissues. This is consistent with its expression profile in prostate cancer, gastric cancer, non-small cell lung cancer, and colorectal cancer tissues [5–9]. However, paired analysis of NCAPD3 expression revealed that it was not consistently highly expressed in all PTC tissues, and the expression levels varied. This indicates that NCAPD3 expression in PTC tissues exhibits strong tissue specificity. Further investigation identified a significant correlation between NCAPD3 expression and key clinicopathological features, including tumor size and lymphovascular invasion. These findings suggest a close association between elevated NCAPD3 expression and aggressive clinicopathological features, implying a potential role of NCAPD3 in PTC progression. However, due to the limited sample size (n = 20) in the current study, these findings require validation in larger patient cohorts. Future research with expanded sample sizes is necessary to clarify the relationship between NCAPD3 expression and clinicopathological parameters in PTC.

Further evaluation of the biological role of NCAPD3 revealed that NCAPD3 silencing inhibited K1 and TPC-1 cell proliferation, migration, and invasion. These results suggested that NCAPD3 promotes PTC progression. NCAPD3 also promoting the tumorigenesis of prostate cancer, gastric cancer, non-small cell lung cancer, and colorectal cancer tissues [5–9]. These studies indicate that the role and expression pattern of NCAPD3 in different cancer types are consistent, suggesting its potential to become a pan-cancer oncogene. However, this speculation requires further research to confirm its roles and mechanisms in a wider range of cancer types.

Abnormal energy metabolism is an important characteristic of cancer cells. Aerobic glycolysis, also known as Warburg effect, is related to the biological behaviors of tumor cell proliferation, migration, and invasion [11–13]. Aerobic glycolytic metabolism is considered a new direction in tumor therapy. LDHA and PKM2 are the key factors in aerobic glycolysis^10^. PKM2 catalyzes the final step of glucose metabolism, converting phosphoenolpyruvate into pyruvate, while generating ATP in the process [14–16]. LDHA is responsible for converting pyruvate into lactate, and a large amount of lactate is generated in tumors to provide an acidic microenvironment, inducing tumor cell proliferation, metastasis, and immune escape [14–16]. In this study, we found that NCAPD3 silencing reduced LDHA, PKM2, and lactate levels. Our results revealed that NCAPD3 may play a promoting role in the aerobic glycolysis of PTC. Based on the key role of aerobic glycolysis in regulating tumor growth and metastasis, we speculate that NCAPD3 may involve in the proliferation and metastasis of PTCby regulating the aerobic glycolysis pathway. However, this speculation needs more experiments to verify.

Our study has some limitations. Firstly, the sample size included in this study is small, which may affect the reliability of the conclusions. Secondly, the relationship between NCAPD3 expression and clinical characteristics as well as prognosis, including overall survival and progression-free survival, in patients with PTC has not been analyzed. Thirdly, an animal model was not established to validate the role of NCAPD3 in the regulation of PTC. Fourthly, the molecular mechanism by which NCAPD3 regulates aerobic glycolysis remains unclear. Finaly, we did not investigate the relationship between NCAPD3 expression and the genetic characteristics of thyroid neoplasms. This study is a preliminary investigation of the function of NCAPD3 in PTC, and further studies are needed to address these issues.

## Conclusion

NCAPD3 was highly expressed in PTC tissues and correlated with aggressive clinicopathological features (e.g., tumor size and lymphovascular invasion), and NCAPD3 silencing inhibited proliferation, migration, invasion, and aerobic glycolysis of PTC cells. These observations suggest that NCAPD3 may be a potential therapeutic target for PTC. Our present study provides new insights for the targeted therapy of PTC.

## Supplementary Information

Additional file 1.

## Data Availability

Data is provided within the manuscript.
