# Supplementary figures and images for: NCAPD3 is involved in papillary thyroid carcinoma proliferation, metastasis, and aerobic glycolytic pathway
Source: Discov Oncol. 2025 May 30;16:955. doi: 10.1007/s12672-025-02767-x (PMC12125433; doi:10.1007/s12672-025-02767-x)

# K1 NCAPD3

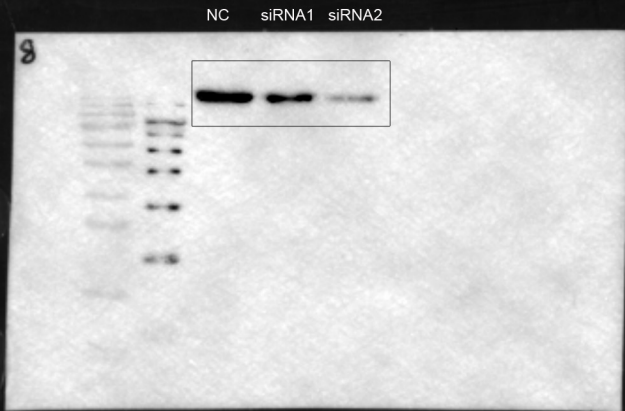

# K1 GAPDH

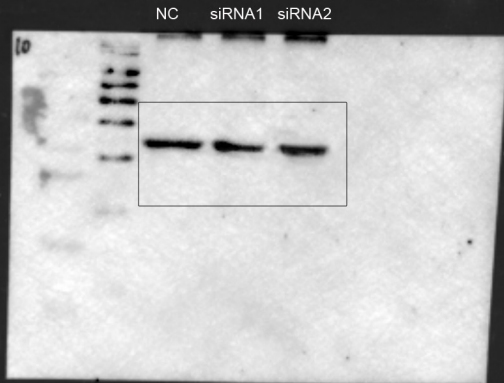

# TPC-1 NCAPD3

NC siRNA1 siRNA2

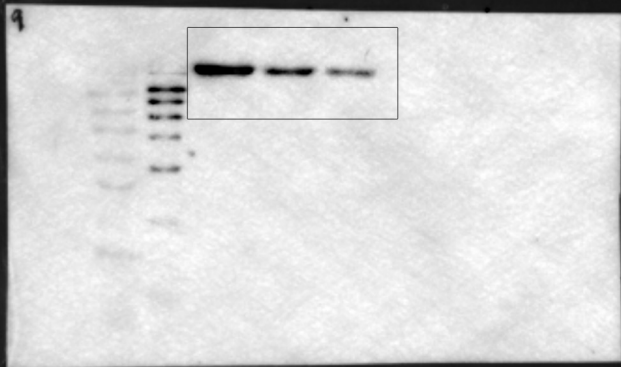

# TPC-1 GAPDH

NC siRNA1 siRNA2

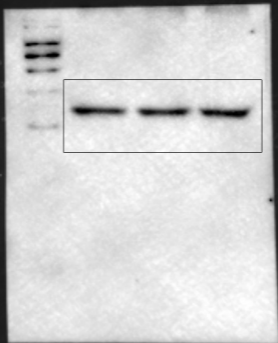

Supplement: Supplementary file 1 — Additional file 1. [file 12672_2025_2767_MOESM1_ESM.pdf]
